# Atomic-Scale Mapping and Quantification of Local Ruddlesden–Popper Phase Variations

**DOI:** 10.1021/acs.nanolett.2c03893

**Published:** 2022-12-06

**Authors:** Erin E. Fleck, Matthew R. Barone, Hari P. Nair, Nathaniel J. Schreiber, Natalie M. Dawley, Darrell G. Schlom, Berit H. Goodge, Lena F. Kourkoutis

**Affiliations:** † School of Applied and Engineering Physics, Cornell University, Ithaca, New York 14853, United States; ‡ Department of Materials Science and Engineering, Cornell University, Ithaca, New York 14853, United States; § Kavli Institute at Cornell for Nanoscale Science, Cornell University, Ithaca, New York 14853, United States; ∥ Leibniz-Institut für Kristallzüchtung, Max-Born-Str. 2, 12489 Berlin, Germany

**Keywords:** scanning transmission electron microscopy (STEM), Ruddlesden−Popper, layered materials, quantitative image analysis, strain mapping

## Abstract

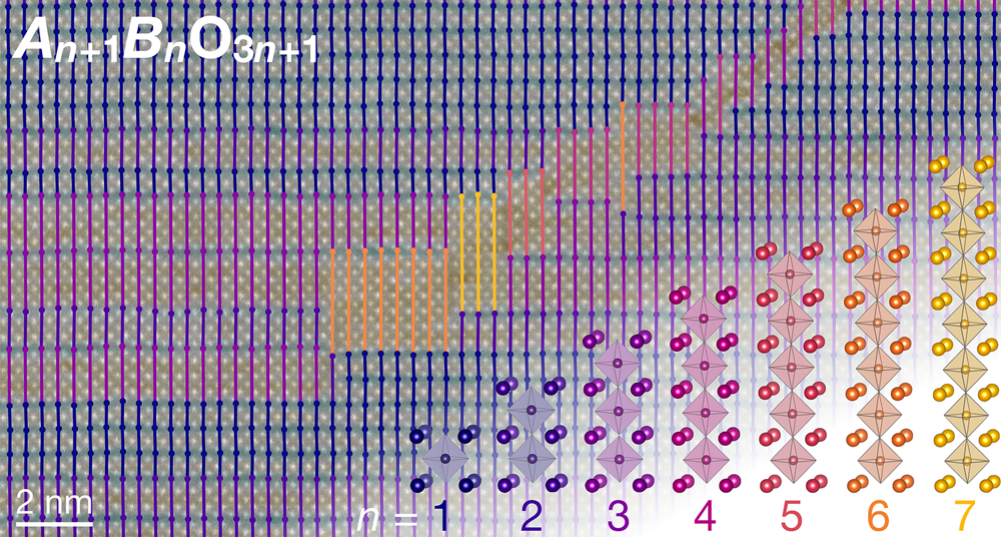

The Ruddlesden–Popper (A_*n*+1_B_*n*_O_3*n*+1_) compounds are highly tunable materials whose functional properties can be dramatically impacted by their structural phase *n*. The negligible differences in formation energies for different *n* can produce local structural variations arising from small stoichiometric deviations. Here, we present a Python analysis platform to detect, measure, and quantify the presence of different *n*-phases based on atomic-resolution scanning transmission electron microscopy (STEM) images. We employ image phase analysis to identify horizontal Ruddlesden–Popper faults within the lattice images and quantify the local structure. Our semiautomated technique considers effects of finite projection thickness, limited fields of view, and lateral sampling rates. This method retains real-space distribution of layer variations allowing for spatial mapping of local *n*-phases to enable quantification of intergrowth occurrence and qualitative description of their distribution suitable for a wide range of layered materials.

Layered materials are characterized by strongly bonded two-dimensional (2D) or quasi-2D atomic planes separated by weaker interplanar bonding which gives rise to anisotropic crystal structures and material properties. The responses of these materials can be tuned by manipulating the dimensionality along the out-of-plane direction. The Ruddlesden–Popper layered perovskite compounds are one such class, described by the general formula A_*n*+1_B_*n*_O_3*n*+1_, where A is an alkali, alkaline earth, rare-earth metal, In, Sn, Pb, or Bi and B is a transition metal.^1−3^ Structurally, *n* is the number of perovskite ABO_3_ layers separated by a single rock salt AO layer, as illustrated by an alternate representation of this formula, (ABO_3_)_*n*_AO. The relative dimensionality of the system is defined by *n*: *n* = 1 phases are strongly anisotropic while *n* = *∞* are three-dimensional perovskites. The atomic structure of an *n* = 6 Ruddlesden–Popper phase is shown in Figure 1a. With careful design and growth, variations between these phases can be exploited to yield a wide variety of functional properties, including colossal magnetoresistance,^4^ superconductivity,^5,6^ and dielectric tunability.^7,8^

**Figure 1:**
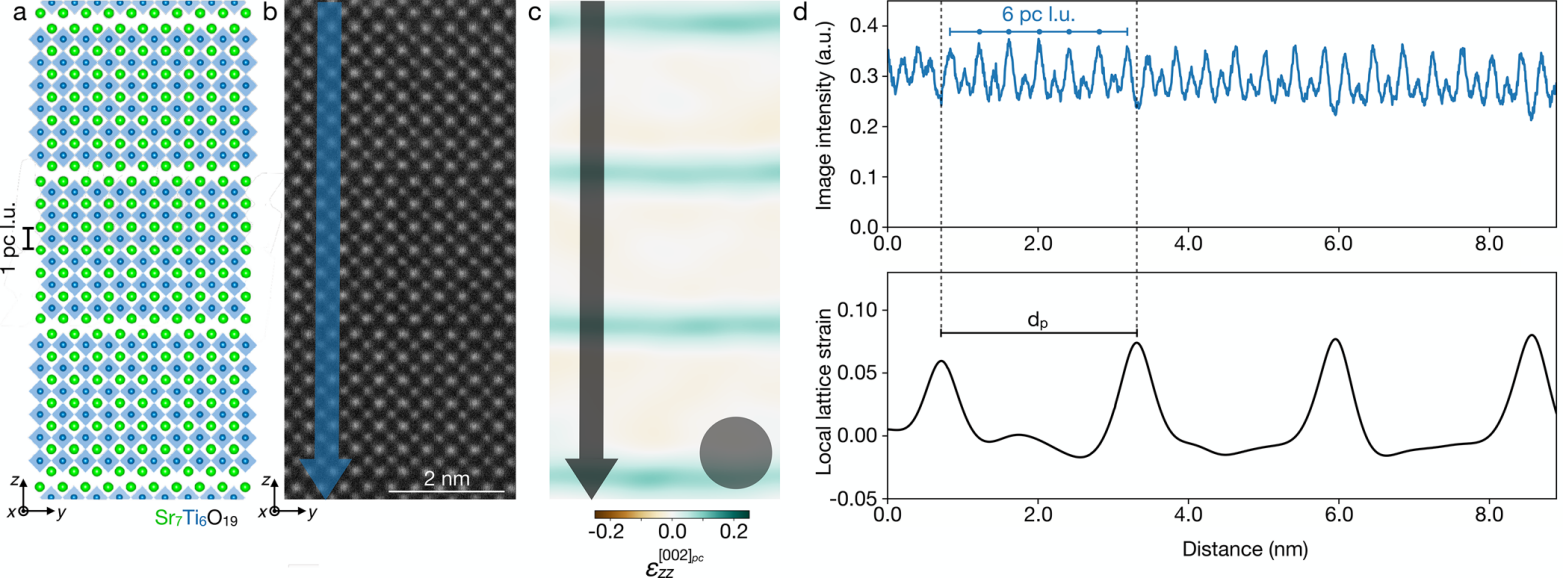
An overview of the counting process for local Ruddlesden–Popper phases. (a) Atomic structure of Sr_7_Ti_6_O_19_, an *n* = 6 Ruddlesden–Popper phase. The pseudocubic lattice unit (pc l.u.) is defined as shown. (b) HAADF-STEM image of Sr_7_Ti_6_O_19_ film. (c) Strain map of the same region as (b) generated by lock-in analysis of the 002*_pc_* Bragg peak. The circle denotes the real-space coarsening length set by the phase lock-in analysis. (d) Top: image intensity averaged over the width of the line shown in (b). Bottom: local lattice strain averaged over the corresponding line in (c). The peak distance, denoted here by *d*_p_, corresponds to the distance between two horizontal Ruddlesden–Popper faults.

The growth of Ruddlesden–Popper compounds—particularly those with higher-*n* phases—becomes difficult to control due to the similar stoichiometry and formation energies among nearby members in a homologous series^9,10^ and can lead to the presence of mixed *n*-phase materials.^11−13^ For some applications these inclusions are inert, but in certain cases they may significantly alter the measured response of the material, even for small variations in *n*. The Ruddlesden–Popper strontium ruthenates, Sr_*n*+1_Ru_*n*_O_3*n*+1_, for example, exhibit remarkably different behaviors at low temperatures: the *n* = 1 phase Sr_2_RuO_4_ is an unconventional superconductor,^14,15^ while the higher-*n* phases (*n* = 3 Sr_4_Ru_3_O_10_, *n* = 4 Sr_5_Ru_4_O_13_, and *n* = *∞* SrRuO_3_) are ferromagnetic metals,^16^ and the ground state of *n* = 2 Sr_3_Ru_2_O_7_ can be pushed to a ferromagnetic state with small perturbations.^17,18^ Particularly for heterostructures that may include multiple such phases carefully chosen for their functional properties, quantifying the occurrence and spatial distribution of different Ruddlesden–Popper layers is therefore of key importance.

Bulk techniques such as X-ray diffraction (XRD) can asses the overall crystallinity of these materials, but quantifying subtle variations in the precise layering structure requires more precise methods of characterization. Using a sub-angstrom electron probe, aberration-corrected scanning transmission electron microscopy (STEM) provides a direct visualization of the atomic lattice and defects or variations therein. In high-angle annular dark-field (HAADF)-STEM imaging, the relative intensity of each atomic column scales approximately as the square of its atomic number, such that heavier species appear brighter and lighter elements dimmer. Figure 1b shows a HAADF-STEM image of the *n* = 6 Ruddlesden–Popper Sr_7_Ti_6_O_19_^19^ in which the layered perovskite motif comprising brighter strontium (*Z* = 38) and dimmer titanium (*Z* = 22) atomic columns is clearly visible, as are adjacent rock salt spacer layers at the horizontal Ruddlesden–Popper faults between each perovskite slab. By cross-sectional imaging, HAADF-STEM can directly identify *n*-phase variations with exceptional sensitivity. To build an accurate representation of a macroscopic sample, however, it is imperative to establish a systematic and statistical method for quantifying such variations.

Here, we present an openly available analysis platform implemented in Python to detect, measure, and quantify the presence of different Ruddlesden–Popper *n*-phases based on atomic-resolution STEM images. We employ phase lock-in analysis^20^ to identify the expanded interplanar distance at horizontal Ruddlesden–Popper faults which appear as regions of high tensile strain. Using these high-strain faults, we identify and quantify local *n*-phases. Repeated analysis over multiple data sets provides statistical characterization of a sample, including quantitative measurements of *n*-phase occurrences and real-space mapping of their distribution.

Fourier analysis is an effective way to extract detailed structural information from periodic frequencies in STEM images. In crystalline materials, strong peaks in the fast Fourier transform (FFT) correspond to spatial frequencies (distances) between high-symmetry planes. Isolating the frequency of a single peak extracts contributions to an image from a chosen set of atomic lattice planes (fringes). Further analysis can detect subtle changes to these periodicities, such as variations in the spacing between atomic planes.^20,21^

Here, we identify the rock salt spacer layers between adjacent perovskite slabs by mapping local lattice strain in the 002_*pc*_ (*pc* = pseudocubic) fringes, which are sensitive to both the AO and BO_2_ planes along the *c*-axis. Strain analysis highlights the horizontal Ruddlesden–Popper faults due to different interplanar spacings between consecutive AO–AO layers at the horziontal faults and AO–BO_2_ layers within a perovskite slab. For example, the pseudocubic *c*-axis lattice constant in perovskite-phase SrTiO_3_ is 3.905 Å, so the (002) plane spacing is ∼3.905 Å/2 ≈ 1.95 Å. At the rock salt spacer layers between adjacent *n* = 6 Ruddlesden–Popper phases, however, the separation between SrO planes is 2.79 Å. Frequency analysis on the 002_*pc*_ peak will thus highlight horizontal Ruddlesden–Popper faults as strong interplanar expansion from ∼1.95 to 2.79 Å, which appears as local tensile lattice strain and yields out-of-plane strain maps like the one shown in Figure 1c. While similar analysis of the 001_*pc*_ peak also identifies Ruddlesden–Popper faults, it is less suited to the methods described here because it additionally highlights vertical Ruddlesden–Popper faults where BO_2_ planes are offset along the *c*-axis and regions of reduced contrast arising from mixed atomic projection in the cross-sectional specimen. The technique presented here can be further extended to other Fourier peaks depending on applications of interest, such as isolating only vertical faults with the 200_*pc*_ peak or identifying both horizontal and vertical faults using the 101_*pc*_ peak.

It is important to note that what we refer to as “strain maps” are measuring local changes to the periodic fringes, which include both real crystalline lattice strains and unstrained effects such as the expansion across the horizontal Ruddlesden–Popper fault. Because this technique is a relative measurement, the total strain across a single image must average to zero such that strong positive strain at Ruddlesden–Popper faults is compensated by small apparent negative strain within the perovskite slabs although there is no change to the interplanar spacing within these regions.

The 002_*pc*_ tensile strain map shown in Figure 1c demonstrates how this analysis generates signatures of high positive strain directly at horizontal Ruddlesden–Popper faults. By converting the two-dimensional image of local lattice strain into a series of line profiles along the crystalline *c*-axis, local maxima in the strain profile identify the vertical position of each fault. As demonstrated in Figure 1d, the real-space distance between two consecutive peaks directly corresponds to the distance between two rock salt spacer layers, denoted here as *d*_*p*_. The measurement of this distance in image pixels is converted into local *n*-phase by calibrating the lattice constant with the FFT.

Because peaks within each line profile fall in the middle of Ruddlesden–Popper faults rather than centered on either of the AO planes, the precise measurement between two strain peaks is a noninteger value of pseudocubic lattice units (l.u.) which overestimates the local *n*-phase. This extra distance can be seen in Figure 1d, where the peaks in the local lattice strain profile fall slightly outside the STEM image intensity profile peaks which correspond to the first and last atomic planes of a Ruddlesden–Popper layer. This excess, however, will necessarily not exceed one pseudocubic lattice unit because the spacing between rock salt spacer layers is smaller than the pseudocubic lattice constant, so the local *n*-phase is obtained by flooring the calculated number of pseudocubic lattice units. This process is repeated across an image to yield quantitative data about the relative densities of different *n*-phases and their spatial distribution.

Our method relies on the ability to clearly identify horizontal Ruddlesden–Popper faults as local maxima in crystalline strain profiles. Strain maps should first be carefully optimized for contrast and real-space coarsening set by the mask size chosen during the Fourier analysis^20^ (Supporting Information Figure S1). We find that a good rule-of-thumb is real-space coarsening between 2 and 4 times the pseudocubic lattice size (e.g., ∼8–16 Å, for the oxide compounds studied here with lattice spacings ∼4 Å). Larger mask sizes may be required for more two-dimensional (lower-*n*) compounds to ensure that Ruddlesden–Popper faults are clearly identified. The Fourier mask sizes and other quantitative parameters used for all analysis presented here can be found in example Python notebooks included in the Supporting Information.

Additional consideration is required to account for the finite projection thickness inherent to cross-sectional STEM imaging. Figure 2a shows a schematic side view of a stacking fault in the plane of the sample. In projection, these stacking faults create regions where two or more Ruddlesden–Popper structures are vertically offset from each other by half a perovskite lattice unit. Mixed projection through both A- and B-site atomic columns results in reduced contrast in standard HAADF-STEM imaging and obscures the clarity of horizontal Ruddlesden–Popper faults compared to cleaner regions of the sample.

**Figure 2:**
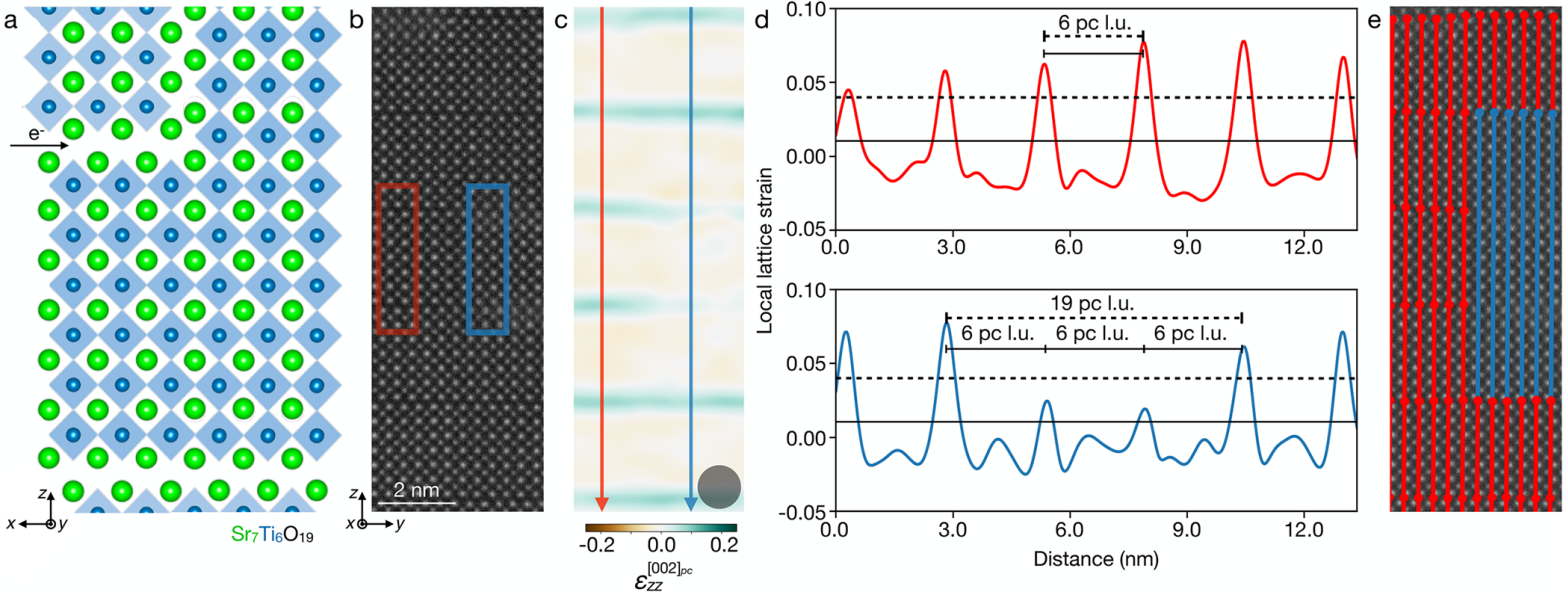
Consequences of STEM imaging projection through cross-sectional specimens of finite thickness. (a) A side-view visualization of the electron beam encountering a stacking fault in a Ruddlesden–Popper phase during STEM imaging, which could give rise to blurred contrast as shown in (b), a HAADF-STEM image of an Sr_7_Ti_6_O_19_ film. The red box highlights a region of the film with distinct rock salt spacer layers, while the blue box highlights the region on the right where contrast in the horizontal Ruddlesden–Popper fault has blurred due to mixed projection. (c) Tensile strain map of the same region as (b) generated by lock-in analysis of the 002_*pc*_ Bragg peak, with the circle again denoting the real-space coarsening length set by phase lock-in analysis. (d) Line profiles of local lattice strain averaged over widths of the red (above) and blue (below) arrows in (c). The solid and dashed lines represent strain thresholds of 0.01 and 0.04, respectively, and the pseudocubic lattice unit counts (pc l.u.) resulting from each. (e) Map of local Ruddlesden–Popper phases in the region shown in (b), where regions of *n* = 6 and *n* = 19 are indicated by red and blue lines, respectively.

Figures 2b and 2c show an area of Sr_7_Ti_6_O_19_ that contains faults which are clear in some portions (red) but blurred elsewhere (blue). In the red region, clear atomic contrast between A and B sites (Sr and Ti) and distinct horizontal Ruddlesden–Popper faults are visible in the HAADF-STEM image. The corresponding strain profile contains obvious strong peaks which can be easily identified as Ruddlesden–Popper faults by their intensities, widths, and spacing (Figure 2d, top).

The rock salt boundaries within the blue box are less clear. The faults do not extend through the full projection thickness of the STEM lamella (∼20–30 nm), similar to the schematic in Figure 2a. The corresponding strain profile across this region (Figure 2d, bottom) has peaks of intermediate intensity clearly above the background noise of the strain maps but significantly lower than at clean horizontal faults. Including or excluding these peaks in the analysis will produce different measurements of the local *n*-phase.

To account for this, we define a minimum peak height to qualify as a “sufficient” horizontal Ruddlesden–Popper fault for structural characterization. For example, in Figure 2d (bottom), a generous local lattice strain threshold of <0.02 classifies this region of the film as three distinct *n* = 6 phases. A higher threshold (local lattice strain ≥0.03) more strictly enforces that sufficient horizontal Ruddlesden–Popper faults extend through the full lamella thickness, classifying regions of mixed projection as the measured *n*-phase between “complete” faults. Here, the three *n* = 6 layers identified by the lower tolerance are instead counted as one *n* = 19 phase (note that the spacing between rock salt spacer layers at a horizontal Ruddlesden–Popper fault allows for one additional (AO)_2_ plane, resulting in *n* = 19 rather than *n* = 6 × 3 = 18).

The threshold should be optimized for each sample and application, particularly for samples with significant regions of mixed projection. In cleaner systems, variable threshold values will yield identical quantitative results (Figure 2d, top). The threshold value should be determined in concert with the strain map Fourier mask size to balance sensitivity and reliability (Supporting Information Figure S1). Appropriate thresholds could also be quantitatively identified from simulated HAADF-STEM images of Ruddlesden–Popper faults at different depths along the projection direction which have been subject to identical strain mapping analysis as the experimental data of interest. It may also be fruitful to extend this analysis more directly along the projection direction with through-focal or depth-sectioned image series acquired at the same sample location to identify the depth of a mixed-projection Ruddlesden–Popper fault.^22^ Experimentally, projection effects can be minimized by careful preparation of high-quality, thin cross sections.

The same strain profile peaks that are used to extract the *n*-phase can be correlated to real-space image coordinates which we use to “map” local *n*-phases represented by differently colored lines, e.g., red for *n* = 6 and blue for *n* = 19 in Figure 2e. This provides a useful visualization tool for statistical analysis as well as detailed qualitative insights of sample growth or heterostructuring.

One consideration that complicates this characterization process is the limited field of view of each image. Unlike in the *n* = *∞* end-member of the Ruddlesden–Popper series, the stacking sequence of finite-*n* Ruddlesden–Popper phases means that imaging and characterization of a sample is not translationally invariant. A naive application of a counting process with this limitation can result in data bias when the edges of a particular Ruddlesden–Popper region fall outside the field of view. This bias increases when the characteristic distance between horizontal Ruddlesden–Popper faults is larger, i.e., for samples with greater prevalence of high-*n* phases. Figure 3 demonstrates how the limited field of view can impact statistical results in an *n* = 20 Ruddlesden–Popper (Sr_0.4_Ba_0.6_)_21_Ti_20_O_61_ film.^9^ The upper-right corner of the field of view shown in Figures 3a and 3b contains a region with local *n* > 20. Characterization of other images of the same sample suggests that this region is likely *n* = 41 or *n* = 61. From this image, however, determining the exact *n*-phase along line segments like the one labeled “*n* > 20” in Figure 3a is not possible because one Ruddlesden–Popper fault falls outside the field of view. We therefore discard this and all other segments along the same profile to prevent a statistical bias toward smaller *n*-phases. We require each full line profile to “start” and “end” on or near the same rock salt plane, thereby constraining our quantitative analysis to only areas which can be fully described without any regions of uncertain *n*.

**Figure 3:**
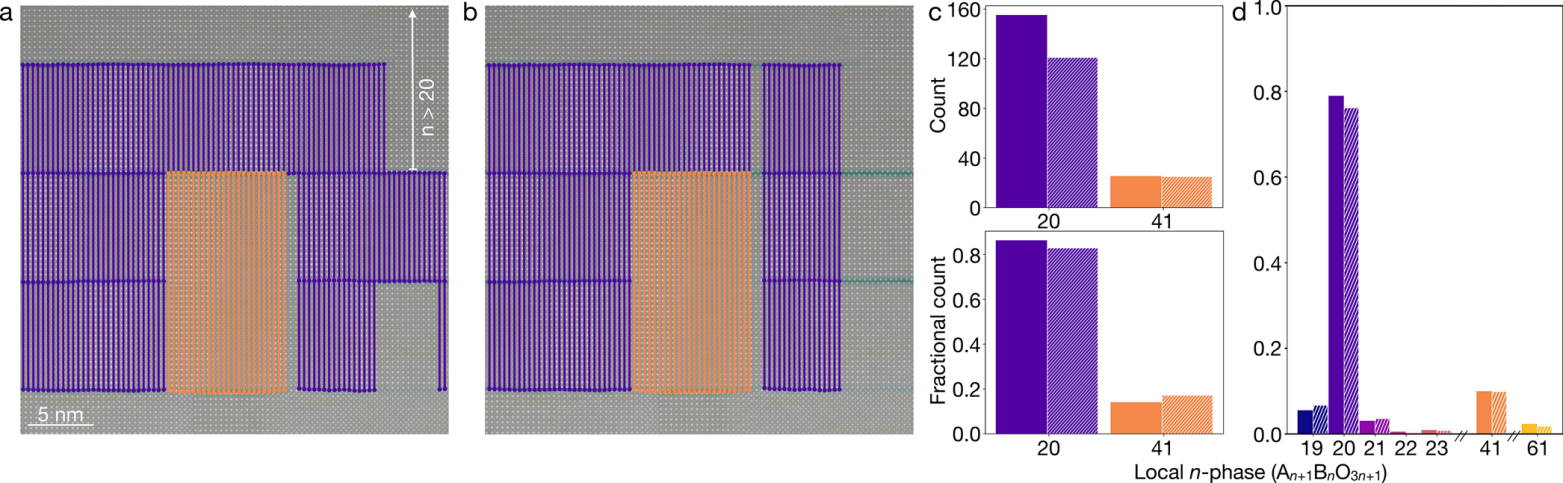
An application of statistical corrections to avoid bias toward smaller *n*-phases on an (Sr_0.4_Ba_0.6_)_21_Ti_20_O_61_ film. (a) Mapping of local Ruddlesden–Popper phases as counted before statistical corrections and (b) after statistical corrections are applied. (c) Top: counts of local Ruddlesden–Popper phases without statistical corrections (solid) and after (hashed). Bottom: the same data displayed as a fraction of total occurrences. (d) Fractional count before (solid) and after (hashed) statistical corrections are applied across a series of several STEM images from different regions of the film. The final result of this analysis was previously reported in Ref. (9).

As illustrated in Figure 3, applying this correction to a single image of an *n* = 20 Ruddlesden–Popper film shows only a slight change in the relative counts of local *n*-phases. The total counts of the *n* = 20 phase (Figure 3c, top) decrease from the uncorrected to the corrected analysis in which weak and out-of-view horizontal faults have resulted in discarded data, such as in the region on the right of Figure 3b. When renormalized by the total number of counts in each case, the effect on fractional counts is less severe (Figure 3c, bottom). We observe a similar trend showing a small reduction in the fractional counts of *n* = 20 regions when this corrected analysis is applied across a larger data set comprising several images of the same sample,^9^ as shown in Figure 3d.

An additional consideration is the horizontal sampling rate. The most accurate analysis uses a sampling rate of every atomic column, as shown in Figures 3a and 3b. The computation time, however, scales with sampling frequency such that sampling at lower frequencies offers an efficient alternative for rapid on-the-fly processing. The maps of local Ruddlesden–Popper phases in a Sr_2_RuO_4_ thin film^23^ shown in Figures 4a and 4b have sampling rates of every 10 and 3 atomic columns, respectively. When the total counts are normalized to a sampling rate of every atomic column, there is very little difference in the distribution of dominant *n*-phases as shown in Figure 4c. For lower *n*-phases (*n* ≤ 3), both the linear and the inset log scale in Figure 4c show similar counts for the sparser (solid) and less sparse (hashed) sampling. With a decreased sampling rate there is, however, some decrease in the precision of the *n*-phase quantification, particularly for infrequently occurring *n*-phases. In this nominally *n* = 1 Sr_2_RuO_4_ sample, the higher *n*-phases (particularly *n* ≥ 4) are counted at different relative occurrences for the two sampling rates, most clearly visible in the inset log scale graph in Figure 4c. Most generally, a denser sampling rate will more precisely characterize the structural phases of a given sample. Here, however, we note that the sparser sampling rate identifies a local *n* = 6 phase while the denser sampling does not. Given the relatively low overlap between the chosen sampling frequencies (every 3 and 10 atomic columns), the two analyses yield subtly different quantitative results while reflecting a similar qualitative picture. Sparse sampling down to 10× less than the ideal therefore offers a relatively fast, computationally inexpensive way to characterize a sample’s microstructure with little loss in accuracy for the dominant *n*-phases.

**Figure 4:**
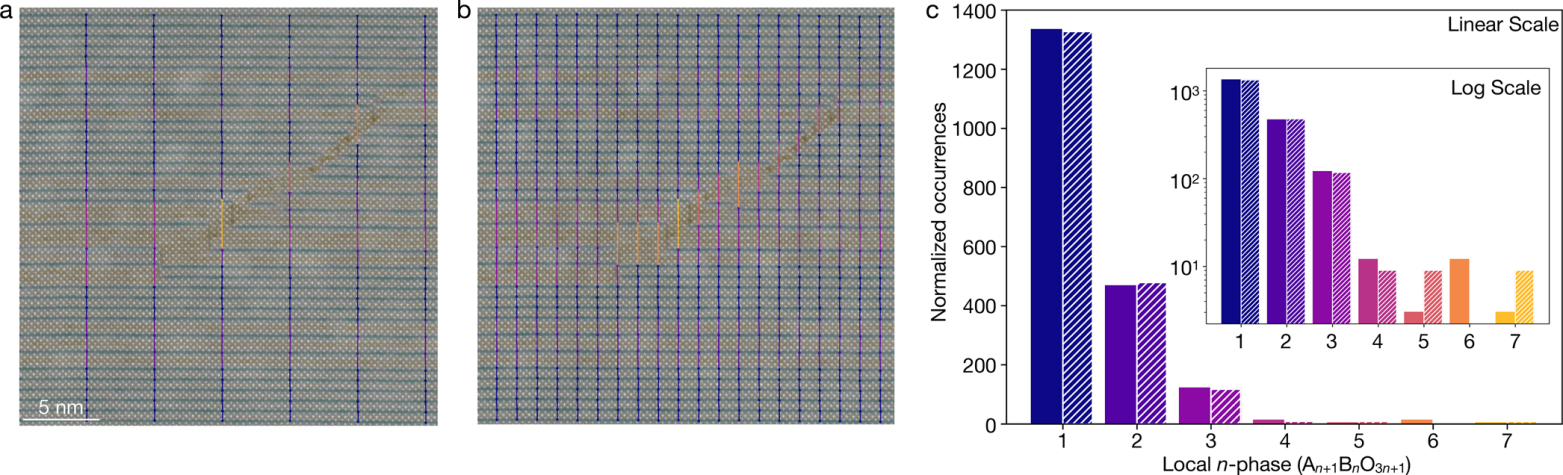
HAADF-STEM image of nominally *n* = 1 Ruddlesden–Popper Sr_2_RuO_4_ with overlays of the local lattice strain map and lines color-coded to represent the identified local *n*-phase with a horizontal sampling rate of every (a) 10 atomic columns and (b) 3 atomic columns. (c) Statistical counts of local Ruddlesden–Popper phase occurrences from (a, solid) and (b, hashed) normalized to an equivalent sampling of every atomic column.

In addition to quantifying different local *n*-phases within a sample, perhaps the most powerful capability of this analysis platform is visually mapping their spatial distribution. Figure 5a demonstrates an optimal analysis (i.e., sampling every atomic column) of the same film as in Figure 4. The histograms of local *n*-phases in Figure 5b show qualitative agreement with those in Figure 4c, as expected.

**Figure 5:**
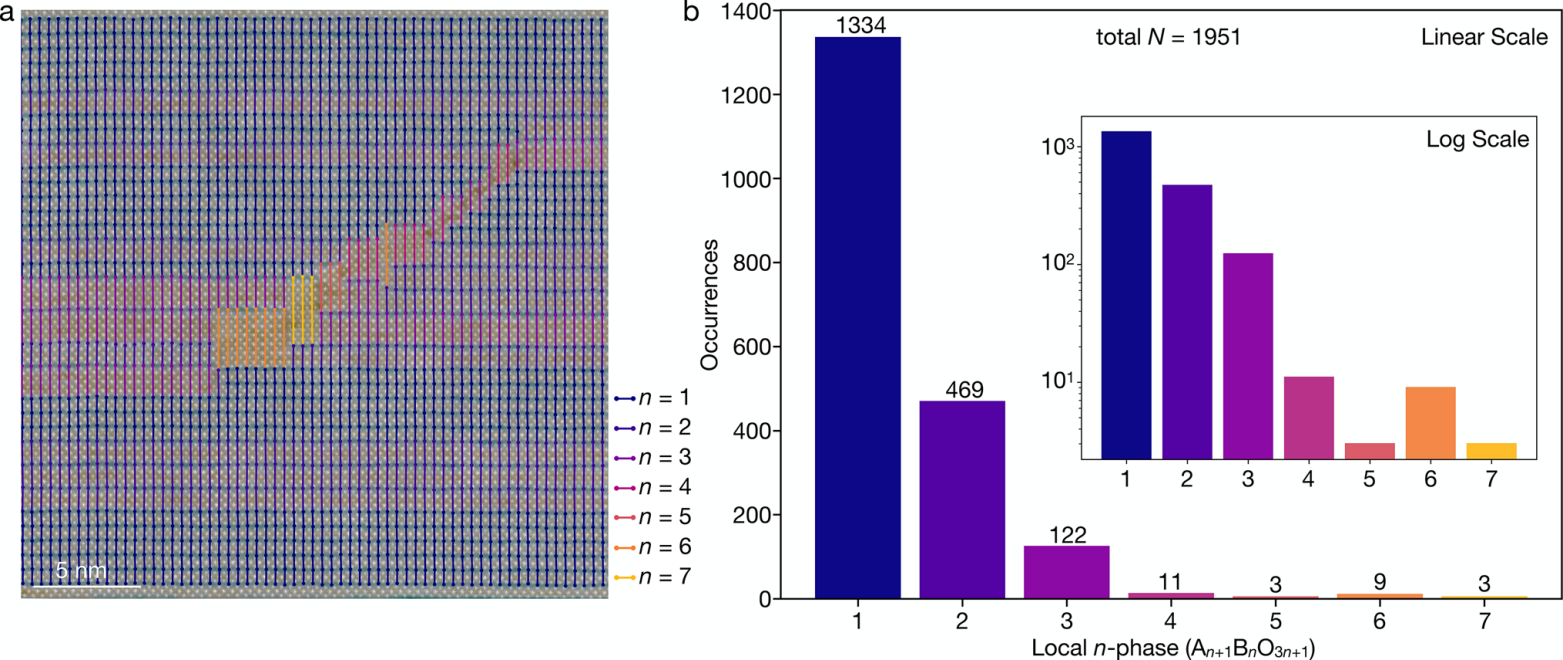
Final results of an ideal sampling mapped onto the same HAADF-STEM image as in Figure 4. (a) HAADF-STEM image of a nominally *n* = 1 Ruddlesden–Popper Sr_2_RuO_4_ film with overlays of the local lattice strain map and lines color-coded to represent the identified local *n*-phase. (b) Statistical counts of local Ruddlesden–Popper phase occurrences in (a) plotted on linear and (inset) log scale.

Deeper insight into the sample growth process can be gleaned from mapping these phase variations in real space. For example, within this film the regions of higher *n*-phases are clustered toward the center of Figure 5a. The remainder of the film visible in this field of view is primarily composed of local regions of *n* = 1 and 2, with a few inclusions of *n* = 3. In this sample, which was grown by adsorption-controlled MBE,^23^ different Ruddlesden–Popper phases reflect local variations in elemental stoichiometry likely related to fluctuating chamber conditions described by the windows of thermodynamic stability for each local *n*-phase.^24,25^ For materials grown by shuttered deposition or other sequential approaches, we can similarly extract information about precise synthesis conditions from variations within the sample. Real-spacing mapping of local *n*-phase distributions thus provides insight into spatial and temporal fluctuations in a material and its synthesis, which can be utilized to modify the growth processes to minimize or exploit defects and intergrowths. Such analysis will be particularly critical for realizing higher *n*-phase Ruddlesden–Poppers^9,10^ as growth becomes more difficult to control with increasing *n*.

At a smaller scale, the atomic reconstructions that form between regions of dissimilar *n* may also have an impact on the local behavior of these layered materials. As previously noted in the discussion of Figure 2d, due to the difference in interplanar spacing for AO–AO layers and AO–BO_2_ layers, “missing” horizontal Ruddlesden–Popper faults do not simply generate a local *n*-phase twice that of the primary phase. Instead, the resulting fault where dissimilar phases meet may result in a local out-of-plane strain. While most thin film growth is concerned with the in-plane epitaxial lattice strain, in certain cases the local out-of-plane strain imposed by heterogeneity in the *n*-phase may play an important role.^26^ In other cases, layers of mixed *n* can be harnessed to help alleviate substrate effects such as step edges.^27^ In regions of high heterogeneity, mapping the junctions between various *n*-phases can provide insight into how local structural phases are formed and arranged within the material.

Understanding the competition or interaction between different phases is a key question for a wide array of Ruddlesden–Popper systems. In layered nickelates, for example, where the structural Ruddlesden–Popper phase governs the formal nickel valence,^6,28,29^ this technique may be a practical way of mapping local electronic variations. Additionally, certain engineered superlattices composed of layers with alternating *n*-phases have been shown to host Heisenberg antiferromagnetism.^30^ The method presented here allows for both quantification and visualization of the syntactic phases in Ruddlesden–Popper (heterostructures) and other layered compounds, paving the way to a deeper understanding of how such variations interact and interface with each other.

## Data Availability

The data that support the findings of this study as well as the developed Python code have been deposited in the Platform for the Accelerated Realization, Analysis, and Discovery of Interface Materials (PARADIM) database: https://doi.org/10.34863/amcp-4s12. This repository includes example notebooks for basic implementation of the phase lock-in method (Goodge, et al. *Microsc. Microanal.***2022**, 28, 404–411) and the Ruddlesden–Popper analysis described here, as well as complete notebooks used to produce the figures in this article with all relevant parameter values (e.g., Fourier mask size, strain threshold, horizontal sampling).

## Supporting Information Available

Examples analysis showing the impact of Fourier mask size and boundary strain threshold, raw output strain maps for Figures 3−5. (PDF)

## References

[ref1] Balz D.; Plieth K. Die Struktur des Kaliumnickelfluorids, K_2_NiF. Zeitschrift für Elektrochemie, Berichte der Bunsengesellschaft für physikalische Chemie 1955, 59, 545–551.

[ref2] Ruddlesden S.; Popper P. New compounds of the K_2_NiF_4_ type. Acta Crystallogr. 1957, 10, 538–539. 10.1107/S0365110X57001929.

[ref3] Ruddlesden S.; Popper P. The compound Sr_3_Ti_2_O_7_ and its structure. Acta Crystallogr. 1958, 11, 54–55. 10.1107/S0365110X58000128.

[ref4] Moritomo Y.; Asamitsu A.; Kuwahara H.; Tokura Y. Giant magnetoresistance of manganese oxides with a layered perovskite structure. Nature 1996, 380, 141–144. 10.1038/380141a0.

[ref5] Müller-Buschbaum H. The Crystal Chemistry of High-Temperature Oxide Superconductors and Materials with Related Structures. Angewandte Chemie International Edition in English 1989, 28, 1472–1493. 10.1002/anie.198914721.

[ref6] Pan G. A.; et al. Superconductivity in a quintuple-layer square-planar nickelate. Nat. Mater. 2022, 21, 160–164. 10.1038/s41563-021-01142-9. 34811494

[ref7] Lee C. H.; et al. Exploiting dimensionality and defect mitigation to create tunable microwave dielectrics. Nature 2013, 502, 532–536. 10.1038/nature12582. 24132232

[ref8] Dawley N. M.; et al. Targeted chemical pressure yields tuneable millimetre-wave dielectric. Nat. Mater. 2020, 19, 176–181. 10.1038/s41563-019-0564-4. 31873229

[ref9] Barone M. R.; Dawley N. M.; Nair H. P.; Goodge B. H.; Holtz M. E.; Soukiassian A.; Fleck E. E.; Lee K.; Jia Y.; Heeg T.; Gatt R.; Nie Y.; Muller D. A.; Kourkoutis L. F.; Schlom D. G. Improved control of atomic layering in perovskite-related homologous series. APL Materials 2021, 9, 021118 10.1063/5.0036087.

[ref10] Barone M. R.; Jeong M.; Parker N.; Sun J.; Tenne D. A.; Lee K.; Schlom D. G. Synthesis of metastable Ruddlesden–Popper titanates, (*A*TiO_3_)_*n*_AO, with *n* ≥ 20 by molecular-beam epitaxy. APL Materials 2022, 10, 091106 10.1063/5.0101202.

[ref11] Venkatesan N. R.; Kennard R. M.; DeCrescent R. A.; Nakayama H.; Dahlman C. J.; Perry E. E.; Schuller J. A.; Chabinyc M. L. Phase Intergrowth and Structural Defects in Organic Metal Halide Ruddlesden–Popper Thin Films. Chem. Mater. 2018, 30, 8615–8623. 10.1021/acs.chemmater.8b03832.

[ref12] Smith I. C.; Hoke E. T.; Solis-Ibarra D.; Mcgehee M. D.; Karunadasa H. I. A Layered Hybrid Perovskite Solar-Cell Absorber with Enhanced Moisture Stability. Angew. Chem. 2014, 53, 11232–11235. 10.1002/anie.201406466. 25196933

[ref13] Calabrese J.; Jones N. L.; Harlow R. L.; Herron N.; Thorn D. L.; Wang Y. Preparation and characterization of layered lead halide compounds. J. Am. Chem. Soc. 1991, 113, 2328–2330. 10.1021/ja00006a076.

[ref14] Maeno Y.; Hashimoto H.; Yoshida K.; Nishizaki S.; Fujita T.; Bednorz J.; Lichtenberg F. Superconductivity in a layered perovskite without copper. Nature 1994, 372, 532–534. 10.1038/372532a0.

[ref15] Maeno Y.; Ando T.; Mori Y.; Ohmichi E.; Ikeda S.; NishiZaki S.; Nakatsuji S. Enhancement of superconductivity of Sr_2_RuO_4_ to 3 K by embedded metallic microdomains. Phys. Rev. Lett. 1998, 81, 3765 10.1103/PhysRevLett.81.3765.

[ref16] Crawford M.; Harlow R.; Marshall W.; Li Z.; Cao G.; Lindstrom R.; Huang Q.; Lynn J. Structure and magnetism of single crystal Sr_4_Ru_3_O_10_: A ferromagnetic triple-layer ruthenate. Phys. Rev. B 2002, 65, 214412 10.1103/PhysRevB.65.214412.

[ref17] Brodsky D. O.; Barber M. E.; Bruin J. A.; Borzi R. A.; Grigera S. A.; Perry R. S.; Mackenzie A. P.; Hicks C. W. Strain and vector magnetic field tuning of the anomalous phase in Sr_3_Ru_2_O_7_. Science Advances 2017, 3, e1501804 10.1126/sciadv.1501804. 28168216 PMC5291698

[ref18] Marshall P. B.; Ahadi K.; Kim H.; Stemmer S. Electron nematic fluid in a strained Sr_3_Ru_2_O_7_ film. Phys. Rev. B 2018, 97, 155160 10.1103/PhysRevB.97.155160.

[ref19] Dawley N. M.; Goodge B. H.; Egger W.; Barone M. R.; Kourkoutis L. F.; Keeble D. J.; Schlom D. G. Defect accommodation in off-stoichiometric (SrTiO_3_)_*n*_SrO Ruddlesden–Popper superlattices studied with positron annihilation spectroscopy. Appl. Phys. Lett. 2020, 117, 062901 10.1063/5.0011136.

[ref20] Goodge B. H.; El Baggari I.; Hong S. S.; Wang Z.; Schlom D. G.; Hwang H. Y.; Kourkoutis L. F. Disentangling Coexisting Structural Order Through Phase Lock-In Analysis of Atomic-Resolution STEM Data. Microsc. Microanal. 2022, 28, 404–411. 10.1017/S1431927622000125. 35190012

[ref21] Hytch M.; Snoeck E.; Kilaas R. Quantitative measurement of displacement and strain fields from HREM micrographs. Ultramicroscopy 1998, 74, 131–146. 10.1016/S0304-3991(98)00035-7.

[ref22] Borisevich A. Y.; Lupini A. R.; Pennycook S. J. Depth sectioning with the aberration-corrected scanning transmission electron microscope. Proc. Natl. Acad. Sci. U. S. A. 2006, 103, 3044–3048. 10.1073/pnas.0507105103. 16492746 PMC1413870

[ref23] Nair H. P.; Ruf J. P.; Schreiber N. J.; Miao L.; Grandon M. L.; Baek D. J.; Goodge B. H.; Ruff J. P. C.; Kourkoutis L. F.; Shen K. M.; Schlom D. G. Demystifying the growth of superconducting Sr_2_RuO_4_ thin films. APL Materials 2018, 6, 101108 10.1063/1.5053084.

[ref24] Nair H. P.; Liu Y.; Ruf J. P.; Schreiber N. J.; Shang S.-L.; Baek D. J.; Goodge B. H.; Kourkoutis L. F.; Liu Z.-K.; Shen K. M.; Schlom D. G. Synthesis science of SrRuO_3_ and CaRuO_3_ epitaxial films with high residual resistivity ratios. APL Materials 2018, 6, 046101 10.1063/1.5023477.

[ref25] Goodge B. H.; Nair H. P.; Baek D. J.; Schreiber N. J.; Miao L.; Ruf J. P.; Waite E. N.; Carubia P. M.; Shen K. M.; Schlom D. G.; Kourkoutis L. F. Disentangling types of lattice disorder impacting superconductivity in Sr_2_RuO_4_ by quantitative local probes. APL Materials 2022, 10, 041114 10.1063/5.0085279.

[ref26] Moore K.; O’Connell E. N.; Griffin S. M.; Downing C.; Colfer L.; Schmidt M.; Nicolosi V.; Bangert U.; Keeney L.; Conroy M. Charged Domain Wall and Polar Vortex Topologies in a Room-Temperature Magnetoelectric Multiferroic Thin Film. ACS Appl. Mater. Interfaces 2022, 14, 5525–5536. 10.1021/acsami.1c17383. 35044754 PMC8815039

[ref27] Kim J.; et al. Superconducting Sr_2_RuO_4_ Thin Films without Out-of-Phase Boundaries by Higher-Order Ruddlesden–Popper Intergrowth. Nano Lett. 2021, 21, 4185–4192. 10.1021/acs.nanolett.0c04963. 33979525

[ref28] Greenblatt M. Ruddlesden-Popper Ln_*n*+1_Ni_*n*_O_3*n*+1_ nickelates: structure and properties. Curr. Opin. Solid State Mater. Sci. 1997, 2, 174–183. 10.1016/S1359-0286(97)80062-9.

[ref29] Pan G. A.; et al. Synthesis and electronic properties of Nd_*n*+1_Ni_*n*_O_3*n*+1_ Ruddlesden–Popper nickelate thin films. Phys. Rev. Materials 2022, 6, 055003 10.1103/PhysRevMaterials.6.055003.

[ref30] Kim H.; Bertinshaw J.; Porras J.; Keimer B.; Kim J.; Kim J.-W.; Kim J.; Kim J.; Noh G.; Kim G.-Y.; Choi S.-Y.; Kim B. J. Sr_2_IrO_4_/Sr_3_Ir_2_O_7_ superlattice for a model two-dimensional quantum Heisenberg antiferromagnet. Phys. Rev. Research 2022, 4, 013229 10.1103/PhysRevResearch.4.013229.

