# Supplementary material for: Atomic-Scale Mapping and Quantification of Local Ruddlesden–Popper Phase Variations
Source: Nano Lett. 2022 Dec 6;22(24):10095–101. doi: 10.1021/acs.nanolett.2c03893 (PMC9801418; doi:10.1021/acs.nanolett.2c03893)
Supplement: Supplementary file 1 — nl2c03893_si_001.pdf [file nl2c03893_si_001.pdf]

Supporting Information for: "Atomic-scale mapping and quantification of local Ruddlesden-Popper phase variations"

Erin E. Fleck,[†] Matthew R. Barone,[‡] Hari P. Nair,[‡] Nathaniel J. Schreiber,[‡] Natalie M. Dawley,[‡] Darrell G. Schlom,[‡,¶,§] Berit H. Goodge,[*,†] and Lena F. Kourkoutis[*,†,¶]

†*School of Applied and Engineering Physics, Cornell University, Ithaca, New York 14853, USA*

‡*Department of Materials Science and Engineering, Cornell University, Ithaca, NY 14853, USA*

¶*Kavli Institute at Cornell for Nanoscale Science, Cornell University, Ithaca, NY 14853, USA*

§*Leibniz-Institut für Kristallzüchtung, Max-Born-Str. 2, 12489 Berlin, Germany*

E-mail: bhg37@cornell.edu.; lena.f.kourkoutis@cornell.edu.

Sample synthesis and preparation

Ruddlesden-Popper thin films were grown by molecular beam epitaxy (MBE) as described elsewhere.[1–3] Cross-sectional STEM specimens were prepared using the standard focused ion beam (FIB) lift-out process on a Thermo Scientific Helios G4 UX FIB or FEI Strata 400 FIB equipped with an Omniprobe AutoProbe 200 nanomanipulator.

STEM image acquisition

HAADF-STEM images were acquired on an aberration-corrected FEI Titan Themis operating at 120 kV ($Sr_7Ti_6O_{19}$) or 300 kV (Sr_2RuO_4 and $(Sr_{0.4}Ba_{0.6})_{21}Ti_{20}O_{61}$) with probe convergence semi-angles of 21 and 30 mrad, respectively. The inner collection angles were 90 mrad ($Sr_7Ti_6O_{19}$), 68 mrad (Sr_2RuO_4), and 56 mrad ($(Sr_{0.4}Ba_{0.6})_{21}Ti_{20}O_{61}$).

Impact of Fourier mask size

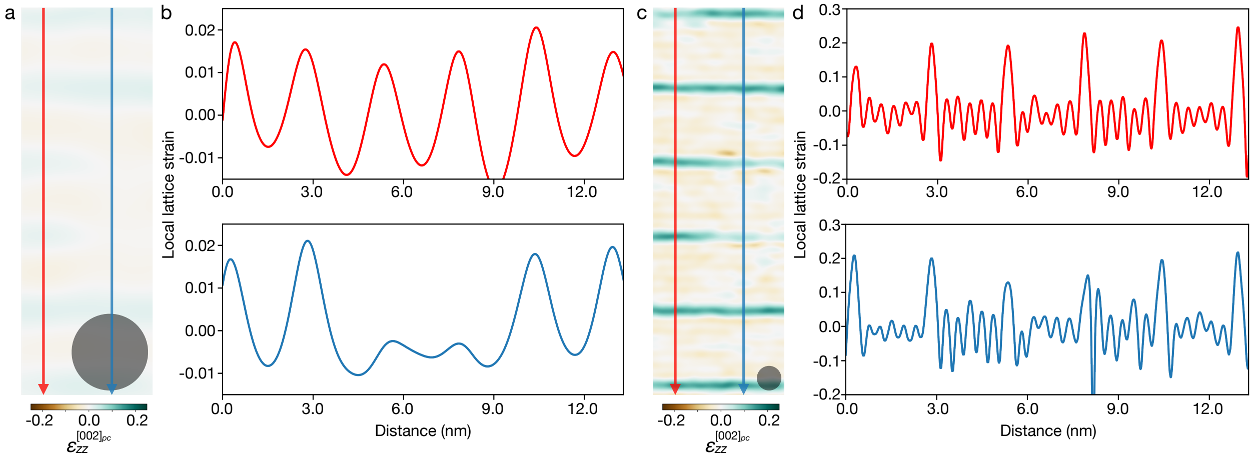

Figure S1: Impact of the Fourier mask size used to generate the strain map on the extraction of Ruddlesden-Popper faults shown for the same region and line cuts as Figure 2 of the main text. (a) A smaller Gaussian mask size results in a larger real-space coarsening, which smooths the strain map and (b) resulting profiles. Weaker or more closely-space Ruddlesden-Popper boundaries may be washed out with small mask sizes. (c) A larger Gaussian mask results in a smaller real-space coarsening, which can enhance the sharpness of peaks in the (d) strain profiles near weak or closely-spaced Ruddlesden-Popper boundaries, but also increases the background variation in the strain profile such that careful thresholding is required. The mask sizes used here are 7, 15, and 22 pixels for the maps shown in (a), Figure 2, and (c), respectively, corresponding to coarsening length scales of 26, 12, and 8 Å as depicted by the black circles on each strain map.

Raw strain map outputs

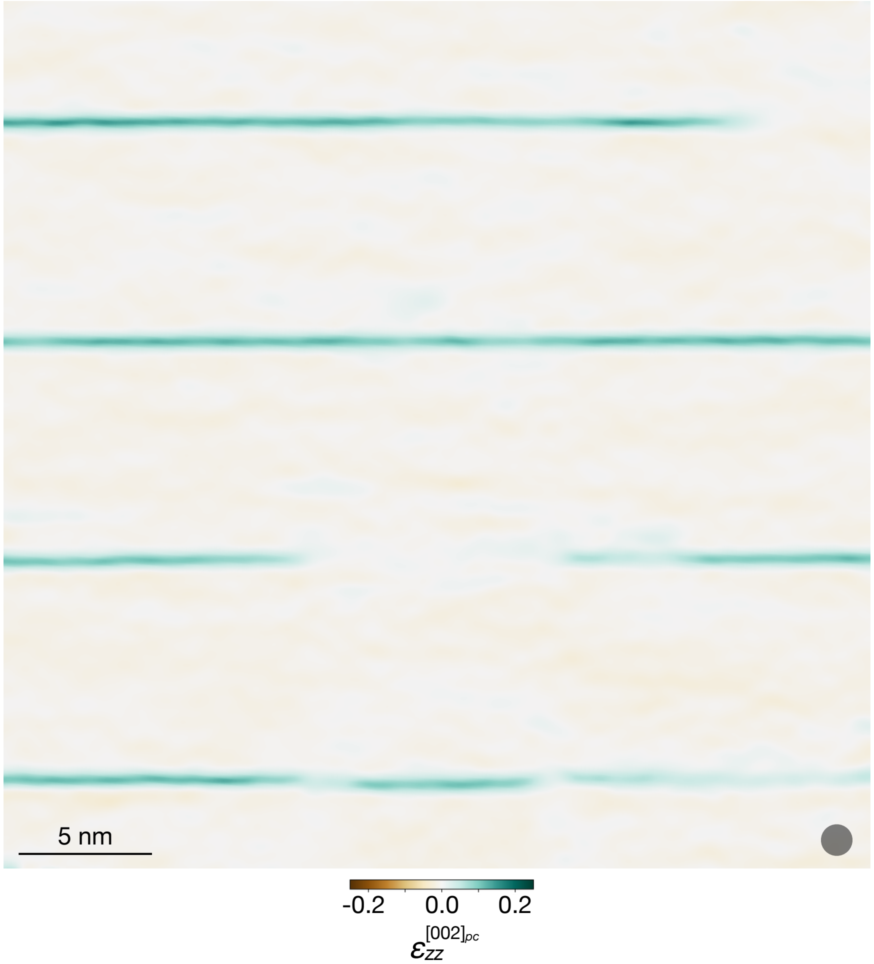

Figure S2: Raw output strain map used for the analysis in Figure 3. The Fourier mask size is 30 pixels, corresponding to a real-space coarsening of 12 Å as shown by the black circle, and the boundary strain threshold is 0.03.

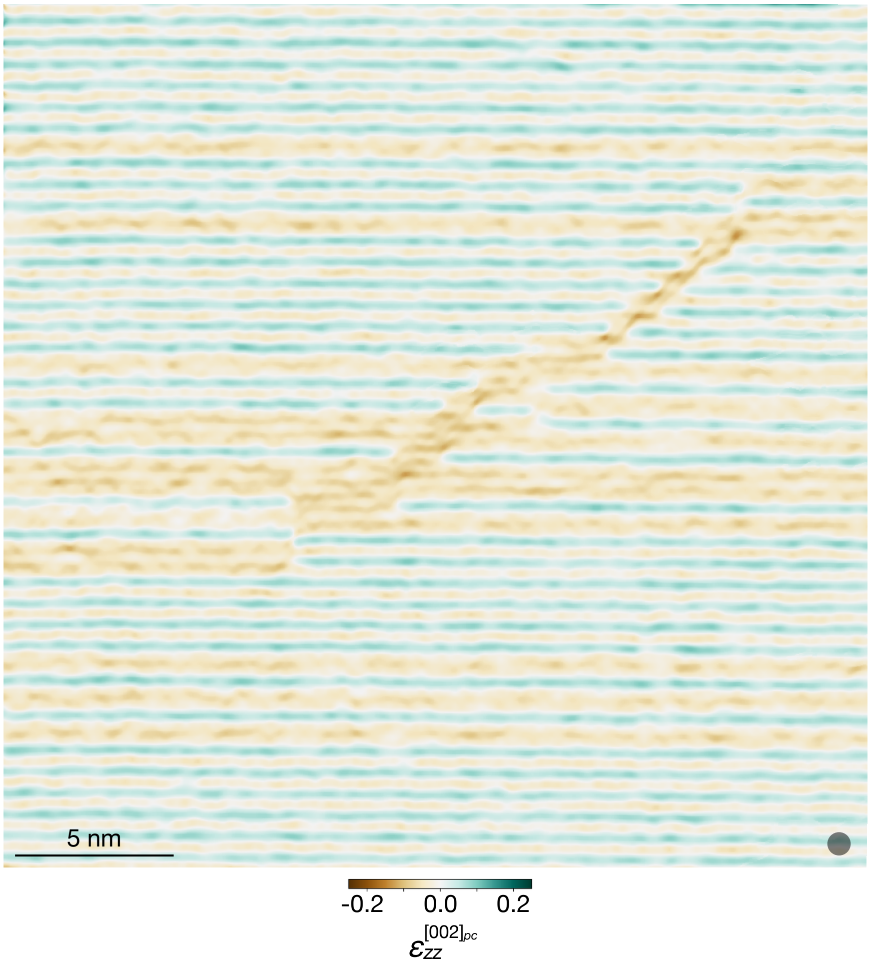

Figure S3: Raw output strain map used for the analysis in Figures 4 and 5. The Fourier mask size is 35 pixels, corresponding to a real-space coarsening of 7.5 Å as shown by the black circle, and the boundary strain threshold is 0.02.

References

(1) Nair, H. P.; Ruf, J. P.; Schreiber, N. J.; Miao, L.; Grandon, M. L.; Baek, D. J.; Goodge, B. H.; Ruff, J. P. C.; Kourkoutis, L. F.; Shen, K. M.; Schlom, D. G. Demystifying the growth of superconducting Sr_2RuO_4 thin films. *APL Materials* **2018**, *6*, 101108.

(2) Dawley, N. M.; Goodge, B. H.; Egger, W.; Barone, M. R.; Kourkoutis, L. F.; Keeble, D. J.; Schlom, D. G. Defect accommodation in off-stoichiometric $(SrTiO_3)_nSrO$ Ruddlesden–Popper superlattices studied with positron annihilation spectroscopy. *Applied Physics Letters* **2020**, *117*, 062901.

(3) Barone, M. R.; Dawley, N. M.; Nair, H. P.; Goodge, B. H.; Holtz, M. E.; Soukiassian, A.; Fleck, E. E.; Lee, K.; Jia, Y.; Heeg, T.; Gatt, R.; Nie, Y.; Muller, D. A.; Kourkoutis, L. F.; Schlom, D. G. Improved control of atomic layering in perovskite-related homologous series. *APL Materials* **2021**, *9*, 021118.
